# Comparability of Self-Ratings and Observer Ratings in Occupational Psychosocial Risk Assessments: Is There Agreement?

**DOI:** 10.1155/2019/8382160

**Published:** 2019-06-12

**Authors:** Isabell Schneider, Martin Mädler, Jessica Lang

**Affiliations:** Teaching and Research Area for Occupational Health Psychology, RWTH Aachen University, Aachen, Germany

## Abstract

**Objective:**

The suitability of self-ratings and observer ratings within organisational management approaches is controversial. The aim of this study was to compare the degree of agreement between self-rated and observer-rated occupational psychosocial demands. The comparison took place within a work-activity and not worker-centred assessment, according to official policies for psychosocial risk assessment. Through simultaneous application of two versions of the same instrument, we aimed to reduce the rating bias to a minimum demonstrating the suitability of self-ratings and observer ratings in companies of all kinds.

**Methods:**

A multimethod online assessment of 22 different work activities was conducted in Germany from October 2016 to October 2017. Workers (self-ratings) and occupational safety and health (OSH) committees (observer ratings) rated the occupational psychosocial risks of each activity with the same instrument (*N* = 669). The instrument measured psychosocial risk conditions at work. Reliability and agreement indices were computed.

**Results:**

The within-group agreement (WGA; *r*_*wg*,*mean*_ =  .42) of the workers' self-ratings was good for each psychosocial risk and the interrater reliability (IRR) was excellent on average (*ICC 2* =  .77) with a medium effect size of* ICC 1* =  .15. The interrater agreement (IRA) between the two groups varied across the activities depending on rating group and activity composition (from *ICC*_*unjust*,*mean*_ =  .39 to *ICC*_*unjust*,*mean*_ =  .86) but was good to excellent on average (*ICC*_*unjust*,*mean*_ =  .71).

**Conclusion:**

The reasonable agreement and excellent reliability in workers' self-ratings justify aggregation of item means at the group level. Furthermore, if the work activities are homogenous and the committee consists of members from different OSH specialties, observer ratings and self-ratings provide comparable results. According to this study's results, both methods are reliable assessment strategies in the context of psychosocial risk assessment. The observer rating approach is especially suitable for small-to-medium enterprises that do not have access to a large anonymous survey assessment.

## 1. Introduction

There is an increasing interest at governmental level (both national and European) in reducing workplace absenteeism and work disability due to adverse (psychosocial) working conditions [1]. A risk assessment, also for psychosocial job demands, is formally standardized in many European countries [2]. However, less than 30% of European companies have implemented measures dealing with psychosocial risks within an organisation-centred management approach [3]. Among those who have, the majority are large enterprises. The strongest drivers of psychosocial risk management are management commitment and employee involvement [4]. Employees can be involved in work councils, OSH committees, or as health and safety representatives. Manager commitment can be encouraged by awareness campaigns. Economic aspects should also be taken into account, for example, by presenting cost-effective assessment approaches. This is because organisations invest less in OSH prevention in times of a recession or economic crisis [4]. This finding is particularly alarming because employees more frequently report psychosocial risks and strain [5, 6] during times of insecure employment. For instance, insomnia ratings were greater among nurses who experienced a pay cut than among nurses whose payment conditions had not changed. [7]. If supervisors were trained in interactional justice (i.e., an intervention aimed at improving psychosocial working conditions), the degree of insomnia and thus the individual strain response decreased faster than for nurses whose supervisors did not receive a training. Thus, the assessment of psychosocial risks during crisis time appears to be a strategic topic [8]. Furthermore, it is essential to involve workers and supervisors in the management process.

The most utilized instruments in identifying psychosocial workplace demands are self-rated questionnaires, because they are inexpensive and easy to quantify and analyse statistically [9]. This has led to a person-centred approach to managing psychosocial risks. However, it is up for debate to what extent self-ratings reflect the objective working conditions [10]. The self-report bias, also known as subjectivity bias, is one of the main concerns regarding self-ratings [11]. Procedures subject to this bias are supposed to be “less objective.” Bias occurs if the characteristics of an individual (e.g., current state of health, expectations, and personality) affect the response of this individual [12]. However, in the context of an organisational management approach to psychosocial risks, it is crucial that measures have an effect as closely as possible on the cause. The main cause is not the individual worker but the working conditions. Therefore, working conditions should be assessed objectively so that the management can react to them appropriately. Objective measures can contribute to a clearer linkage between the subjective perception and the activity conditions [13].

Observation-based assessments are argued to be “more objective” than self-ratings. Observer ratings carried out by OSH experts have three advantages over worker self-ratings [14]. First, due to their years of experience in observing work activities, experts (e.g., occupational health physicians, health and safety experts, and industrial and organisational psychologists) are familiar with the psychosocial conditions of different activities in different companies. Second, as they do not have authority to issue directives to workers, they might be more neutral in their observation as are personnel managers and supervisors. Third, in cases where joint OSH committees of experts and management teams rate working conditions, they might reduce rating bias of supervisors and employees. In addition, since large anonymous surveys require a higher participation rate, to ensure the anonymity of employees, observer ratings are better suited to small and medium sized companies, which lack the amount of workers for an anonymous survey report on their work-specific psychosocial demands. Despite these advantages, observer-ratings have rarely been used to assess psychosocial working conditions [14]. The reason for their scarce use might be that existing instruments are not user friendly, but time consuming, difficult to conduct and interpretation requires the knowledge of industrial and organisational psychologists [15].

In relation to item formulation, the biggest difference between both methods is that observer ratings are formulated in the third-person perspective (e.g., PsyHealth [16]; e.g., “the activity requires […]” or “within the activity it is […]”). On the other hand, self-ratings are presented in the first-person perspective (e.g., Work Design Questionnaire, WDQ [17]; e.g., “the job allows me […]”). Comparative analyses between self-ratings and observer ratings reveal high associations between both methods for job demands that can be observed (e.g., items referring to task complexity, decision latitude, and work environment), whereas low associations have been found for job demands that are less easy to observe and temporally unstable (e.g., items asking about responsibility and time pressure) [18]. Different explanations are possible. In addition to subjectivity bias, the observability of job demands and theoretical conceptualization are mentioned as reasons for differences [19]. For instance, if job demands are conceptualized, in items like “due to the high volume of work, there is a high time pressure,” the person-centred interpretation of items and not the work-related demands are assessed [12]. For these reasons, we argue that within-group agreement is a suitable criterion to evaluate if self-ratings are subjected to the subjectivity bias. A high degree of agreement is a prerequisite for grouping individual values to form a group average [20]. Furthermore, it is suggested by the literature that “conditions (e.g., task conflicts, work interruptions, multitasking, etc.) leading to high job demands are observable, and they might be more appropriate for observation-based measures” [19, p. 198]. We agree that you cannot observe every demand at every time for any work activity, but you can ask experts to rate the demands. We attribute the expert role not only to the employees but also to the OSH experts who also have experience with the activity and the operational procedures. This statement is supported by a meta-analysis of job analyses comparing data sources, workers, analysts, and technical experts for instance. The results demonstrate that, as a data source, workers were less reliable than analysts [21]. Another meta-analysis on job analyses has shown that the number and the time of experience of evaluators are important for reliability [22]. Observer ratings are reliable, if experienced professionals evaluate work activities based on observation and not only on job descriptions [22]. Furthermore, if nonprofessionals carry out the ratings, with a minimum number of 2 to 4 evaluators, a reliability coefficient of .80 is obtained. Overall, a mean reliability around .60 has been identified [21, 22].

Currently, there is no method guaranteeing “objective” measurement [23]. Whether the evaluator is an expert, manager, or worker, there will always remain a rater bias due to the emotional and cognitive evaluation of responses [24]. However, there are methodological solutions to improve reliability and validity of ratings. Scholars have demonstrated that questionnaires with items that are fact-based reduce subjectivity bias and enhance the convergence between self-ratings and observer ratings. For instance, Spector and Fox (2003) minimized the subjectivity bias in the assessment of autonomy by designing scales in which items asked more fact-based and focused questions. In order to test convergent and discriminant validity, they asked workers and supervisors to rate the autonomy of the same job with their new autonomy scale (Factual Autonomy Scale, FAS) and with the autonomy scale of the Job Diagnostic Survey (JDS). FAS ratings of workers and supervisors correlated significantly (*r*=.53,* p*>.05) [25]. If one wants to assess psychosocial working conditions, fact-based items with reference to the working conditions are preferable. The conditions are of key interest, since occupational risks should be prevented at their source [26]. Condition-related self-ratings of the workers as well as condition-related observer ratings are possible methods [12]. Existing instruments that have a self-rating and observer rating version (e.g., ISTA [18]) differ in relation to the perspective of the item formulation and the item numbers. They are not identical in both versions.

Considering the advantages and limitations of both methods, the simultaneous use of observer ratings and workers' self-ratings seems to be a promising strategy for an accurate assessment of psychosocial demands in psychosocial risk assessments [27]. Therefore, the present study describes the comparability of the results of an economic occupational psychosocial risk assessment presented as a self-rating and observer rating version.

Through analysing the comparability of self-ratings and observer ratings, the aim of our study is to promote more objective advances in measuring psychosocial demands within a work-centred approach. We operationalized comparability with different agreement measures for absolute consensus between different raters and reliability with measures for relative consistency of the rank order [28]. We first wanted to know if workers agree on the frequency of psychosocial work. Agreement determines whether the rating of one individual worker corresponds to the ratings of the other workers with the same activity. Second, we wanted to know whether agreement depends on the affiliation to the work activity rated. In other words, we analysed whether the activity explains individual differences in the workers' responses. If these two criteria are fulfilled, self-ratings are reliable sources and suitable measures for risk assessments; thus the subjectivity bias is negligible. Furthermore, in the third step, we wanted to know whether the results of the worker's self-ratings are comparable to observer ratings of OSH committees. This finding would further stress the point that risks can be collected independently of the rater. Furthermore, it promotes a multidisciplinary management approach that takes different perspectives into account by involving different organisational specialties (e.g., staff council representatives, supervisors, occupational safety, and health experts).

We formulated the following hypotheses. Our first hypothesis is that workers of the same work activity rate psychosocial demands with good agreement (*hypothesis 1)*. The second hypothesis is that the workers' self-ratings are reliable (*hypothesis 2). *Third, we hypothesise that the average agreement between workers' self-ratings and observer ratings of the same work activity is good* (hypothesis 3)*.

## 2. Materials and Methods

We collected the data during a two-year cooperation project between the study centre and a social accident insurance. The study was advertised by the social accident insurance in their membership magazine. The participants were thus jobholders of those companies. PhD projects delivered additional data from the local area of the study centre. Data was collected with a self-programmed software [29] from October 2016 to October 2017 via the online instrument PsyHealth [16].

### 2.1. Participants

The sample consisted of two rating groups: self-ratings of workers (*N* = 598) and observer ratings of occupational safety and health (OSH) committee members (*N* = 71). Each group rated the same activity within their respective organisation. Overall, 22 different activities were rated in 11 different organisations. The activities ranged from administrative tasks in the service sector to manual activities in production. You can find an overview of all activities assessed in the present study in the first column of Table 2. For privacy protection within the companies, all self-rating groups consisted of at least 10 workers. The composition of each OSH committees varied. In most cases, committees included supervisors, staff council representatives, safety representatives, occupational physicians and safety officers, and representatives of the human resource department. Table 2 provides also an overview of the individual committee composition for each activity (see the notes of Table 2).

### 2.2. Procedure

The occupational psychosocial risk was assessed with the instrument PsyHealth, a custom-built software solution for online assessment of psychosocial work conditions. The instrument has been designed as a tool for psychosocial risk assessment for both workers' self-ratings and committee observer ratings. For 48 items, participants have to indicate how often each psychosocial working condition occurs while conducting the work activity. The response scale ranges from 0 (“at no time or some of the time”) to 3 (“most or all of the time”). Some items have been reverse-coded in order to avoid response bias. All items are formulated condition-related and are coded in a way where higher values represent better working conditions. The items and response scales are identical for both versions. Thus, the degree of agreement clearly depends on the raters and not on the number of items or perspective. That is why PsyHealth is particularly suitable for analysing comparability of self-ratings and observer ratings.

The invitation to the survey was sent by e-mail with a link to the software. Jobholders and observers received different access codes and were matched by company and name of activity. In order to guarantee the anonymity of the participants and to foster trust, we have not assessed any personal data. Prior to the online assessment, all participants gave their informed consent to their participation in the study. Participation was voluntary. No ethical statement was necessary since we did not collect any sensitive data and data collection was completely anonymous (the codes for company workers were identical for each company, so that it was not possible to track an individual response back to the worker).

### 2.3. Statistical Analyses

For testing our hypotheses, we used the package multilevel 2.6 [30] in R Version 3.3.3 [31]. The multilevel package provides agreement and reliability measures representing the variance in any worker's response that might be explained by the activity.

To test* hypothesis 1* we calculated *r*_wg_ [32] as a measure of within-group agreement (WGA) of self-ratings on the item level. *r*_wg_ determines whether the work activity rating of one individual corresponds to the ratings of the others with the same work activity. Dunlap et al. (2003) showed that the 95% confidence interval for the single item *r*_wg_ varies as a function of group size and the number of response options [33]. We provided the appropriate cut-off values for the current assessment with a four-point frequency scale and an average group size of 27 raters. Based on 10,000 simulations .22 is the 90% confidence interval (*CI*) estimate for low agreement, and .28 is the 95%* CI* estimate for good agreement. The 99% confidence interval value indicating very good agreement is .38.

For testing* hypothesis 2* intraclass correlation coefficients (ICC) 1 and 2 (*ICC 1 and ICC 2*) from ANOVA models were computed.* ICC 1* values may be interpreted as an effect size estimate. According to LeBreton and Senter [28], small effects are indicated by values around .01, medium effects by .10, and large effects by .25. The* ICC 2* values represent the reliability of group means [20]. Fleiss [34] gives the following interpretations:* ICC 2* < .40, bad;* ICC 2* from .40 to .75, appropriate to good; and* ICC 2* from .75 to 1.00, excellent agreement.

In order to evaluate the comparability of the two methods (*hypothesis 3*), the interrater agreement (IRA) between the self-ratings and observer ratings of two rating groups is of key interest. We computed unjusted* ICC*s of the mean for the mean of each pair of ratings (*ICC*_*unjust*,*mean*_) using IBM SPSS Statistics 25 [35] in order to test the absolute agreement between the two rating methods.

## 3. Results

In line with* hypothesis 1*, the current results suggest that there is significant agreement between workers with the same working activity for 96% of all items. There is no agreement for two items, one referring to “retreat possibilities” and the other referring to “varied postures”. On average, the agreement is good (*r*_*wg*,*mean*_ = .42). The second column of Table 1 presents the agreement values between ratings of the workers with the same activity.

In line with* hypothesis 2 *on interrater reliability of self-ratings, the results indicate an excellent reliability value (*ICC 2* = .77) and a medium effect size (*ICC 1* = .15) across all items. For all but one item (“authority for those responsible”), the reliability values are above the critical threshold. A total of 29 items (61%) show excellent reliability; 18 items (38%) show appropriate reliability.* ICC 1* values vary across the different items ranging from small effects (e.g., a value of* ICC 1 =*.02 for “authority for those responsible”) to large effects (e.g., a value of* ICC 1 =*.49 for “fixed location”). In summary, 18 items (38%) show small effects, 21 items (44%) indicate medium effects, and nine items (19%) suggest large effects. The third and fourth columns of Table 1 present the interrater reliability values.

In individual assessment scores, there is considerable individual-level variability. In spite of that, the working activity influences a substantial proportion of variance in the worker's self-ratings, although it does not alone account for the variability. The results indicate that the work activity is a medium size predictor of individuals' responses within psychosocial risk assessments. According to these results, single ratings of any worker are not a reliable source. However, the group averages are reliable measures. Moreover, the worker's agreement demonstrates that the raters are “interchangeable,” indicating that the subjectivity bias is low and might be neglected.

Regarding* hypothesis 3* on agreement between the different methods, we report a good IRA (*ICC*_*unjust*,*mean*_ =  .71) on average. For eleven activities (50%), the interrater agreement values are excellent, ranging from *ICC*_*unjust*,*mean*_ =  .77 to *ICC*_*unjust*,*mean*_ =  .86. For ten activities (45%), the IRA is good, ranging from *ICC*_*unjust*,*mean*_ =  .55 to *ICC*_*unjust*,*mean*_ =  .75. For one activity, the IRA value is below the critical threshold; those are “production, service, and stock” (*ICC*_*unjust*,*mean*_ =  .39). Table 2 illustrates the agreement values between the two methods in its last column.

## 4. Discussion

In order to verify objective conceptualization and measurement of psychosocial working conditions, the agreement and reliability of self-ratings of psychosocial working conditions were identified. To judge the comparability of self-ratings and observer ratings in the context of psychosocial risk assessment, the agreement between the two methods was analysed.

Group means of workers' self-ratings are reliable estimates with significant agreement. The average reliability was higher compared to meta-analyses on the interrater reliability (IRR) of job analysis [21, 22]. The item relating to “authority for those responsible” is the only item that is not assessed reliably at the group level. This may be because some activity groups consisted of workers from different hierarchical levels. Although employed managers were assessed as a separate group, group leaders or persons in comparable positions of authority were part of the workers' ratings, leading to inconsistent results, because they may perceive the presence of authority differently from workers without any responsibilities for subordinates.

To conclude, the results strongly suggest the use of worker's self-ratings, whereby results should be interpreted at group level. Besides that, good agreement was achieved by using condition-related items formulated in the first-person perspective. The agreement was higher than studies using a comparable design but items from the first person perspective in the self-ratings [18].

Since most of the currently available instruments use person-centred items with self-ratings in the first-person perspective, the current findings might be limited due to methodological differences in our item formulation. Future research might compare condition-related items with first- and third-person perspectives of the same instrument in order to further investigate the subjectivity bias in self-rating. However, we strongly suggest the general use of condition-related items in research and practice as it resulted in comparable outcomes according to the present study, especially if the third-person perspective is used.

There was no within-group agreement between the workers' self-ratings for the items referring to “retreat possibilities” and “varied postures.” One explanation might be that the working conditions are not the same for all people rating the same activity. For instance, some might have a single office and others an open-space office; some might be able to change their body posture frequently, while others may be required to remain at their desk except for during their lunch break. Both conditions are, however, very important to protect workers' mental and physiological health. Studies have already demonstrated that not only recovery from work stress during nonwork time is important to reduce mental and physiological strain [36] but also at-work recovery exercise can help to enhance concentration and is associated with less fatigue [37]. In relation to varied postures, there is evidence that interventions are able to reduce sedentary behaviour and increase physical activity [38]. Furthermore, interrupting the time spent sitting at the workplace might produce long-term reductions in blood pressure [39].

The comparison of workers' self-ratings and committee observer ratings shows that there is strong agreement between both methods. The agreement between the two methods is higher than what could have been expected from the results of studies with comparable instruments that demonstrated correlations around .53-.54 or lower [18, 25]. Based on our present results, we advise the use of fact-based and condition-related items in both versions for future research and practice. Intriguingly, according to our data, the workers' ratings did not always indicate fewer resources than the OSH committee (e.g., administrative work A in company A, medical-psychological work in company C, and childcare in company G). This additional finding underlines the advantage of fact-based items in relation to objectivity.

For one work activity conducted in production, service, and stock, the agreement between the two rating methods was not as high as for the other activities. One reason might be that the assessment of this activity differed from the assessment of the other activities in the way that the activity group was inhomogeneous, since it contained workers of three different areas of activity. For anonymity reasons, throughout the study, results of the psychosocial working conditions were only generated if at least ten workers rated one activity. Therefore, in company F, the working areas had to be aggregated. This practical issue of aggregation of work activities for the purpose of survey assessment is a problem that may often occur especially with small companies. Based on our results, we cannot recommend aggregating inhomogeneous activities. A better solution might be to assess each activity separately using a different method than a survey. The other reason for low agreement between the two assessment methods in this specific work activity might be that the committee only consisted of two supervisors. The low number of evaluators and/or the lack of diversity in the committee might be additional reasons for the lower agreement. This conclusion is also drawn by other studies which recommend a higher number of experienced raters [22].

However, agreement values of other activity ratings were still good, although the committees were less diverse and consisted of only two evaluators (e.g., pharmaceutical work in company B). Also, activities that were inhomogeneous, but rated by a diverse committee, reached acceptable agreement values (e.g., service, kitchen, technology, and cleaning work in company C). Relating to our results, we are unable to determine conclusively whether homogeneity of activities or member number and diversity within committees are the more significant factors for agreement. Through systematic manipulation of the homogeneity of the activity being rated and the variety of the committee, future research might find out whether the inhomogeneous activity or the limited observer variety is more associated with disagreement. Furthermore, it would be interesting to know if observer trainings on psychosocial work demands (e.g., for executives) might further improve agreement [19].

It is important to consider that committee compositions varied considerably in our study, which might be a limitation of our study. In the end, we are not able to isolate single effects of different committee compositions. However, if anything, we see this as a strength of the study. For each activity, we have tried to find the best possible variant that fits the organisational conditions. We support this approach for the practical application of psychosocial risk assessments in the future. It allows a certain flexibility to adapt the procedure to the organisational conditions and thus increases user-friendliness and acceptance. Future research might focus on the agreement within different committee-rating compositions to derive a more accurate recommendation.

Other limitations of our study are that we relied only on companies in Germany and only companies took part, which already have a structured occupational health and safety system. We would like to further investigate the agreement in companies outside Germany and with other occupational health and safety structures. In addition, the fact that similar approaches may exist in other nations, but that we are not aware of, cannot be ruled out.

According to our study, results of self-ratings and observer ratings of psychosocial risk are comparable if certain aspects are taken into account in their implementation: In order to assess psychosocial working conditions independently from the individual, items should be formulated as condition-related and in the third-person perspective. Furthermore, homogeneous activities should be rated and the committee should consist of OSH specialists as well as workers' representatives.

## 5. Conclusion

As far as we know, this is the first study comparing self-ratings and observer ratings of an instrument for psychosocial risk assessment which consists of identical items and perspectives in both versions. The results have political and practical implications as they justify the application of both methods. Experts now have a scientific justification for the use of self-ratings and observer ratings in the management of occupational psychosocial risks. Moreover, our study shows that a psychological risk assessment with worker participation is possible for every type of company. For companies that are too small for a risk assessment based on large anonymous surveys and cannot afford comprehensive assessment by external professionals, the committee-rating method provides a reliable alternative for conducting psychosocial risk assessment. For all other companies, we advise a simultaneous assessment with self-ratings and observer ratings to emphasize objectivity of the findings. Of course, they could continue to rely exclusively on self-reports, but the involvement of workers, supervisors, and experts into this process might lead to a fairer treatment approach. By demonstrating comparability of self-ratings and observer ratings in psychosocial risk assessment, we hope to foster objective organisation-centred approaches.

## Figures and Tables

**Table 1:** Agreement and reliability estimates of the self-ratings.

*Within-group agreement*		*Interrater reliability*		
Psychosocial risk items	**r**_**wg**_	***ICC 1***	***ICC 2***	***F* ratio**
*Work content*				
Task completeness	.43^*∗∗∗*^	.05	.55	2.22^*∗∗*^
Task variety	.52^*∗∗∗*^	.21	.88	8.11^*∗∗∗*^
Task significance	.63^*∗∗∗*^	.21	.88	8.13^*∗∗∗*^
Influence on task content	.29^*∗∗*^	.20	.87	7.46^*∗∗∗*^
Influence on task execution	.36^*∗∗*^	.27	.91	10.93^*∗∗∗*^
Influence on work pace	.36^*∗∗*^	.10	.74	3.80^*∗∗∗*^
Unambiguous work orders	.46^*∗∗∗*^	.07	.67	3.08^*∗∗∗*^
Clearly assigned responsibilities	.54^*∗∗∗*^	.05	.60	2.51^*∗∗∗*^
Authority for those responsible	.55^*∗∗∗*^	.02	.36	1.6
Skill utilization	.70^*∗∗∗*^	.13	.79	4.86^*∗∗∗*^
Qualification opportunities	.20^*∗*^	.14	.81	5.22^*∗∗∗*^
Advancement opportunities	.27^*∗*^	.13	.78	4.62^*∗∗∗*^
No suppression of emotion	.34^*∗∗*^	.09	.73	3.65^*∗∗∗*^
No critical life events	.44^*∗∗∗*^	.30	.92	12.39^*∗∗∗*^
No aggression/violence	.61^*∗∗∗*^	.31	.92	12.42^*∗∗∗*^
Fixed location	.46^*∗∗∗*^	.49	.96	26.13^*∗∗∗*^
Job security	.30^*∗∗*^	.05	.58	2.35^*∗∗∗*^
Work-life balance	.42^*∗∗∗*^	.15	.83	5.12^*∗∗∗*^
*Work organisation*				
Compliance with working hours	.45^*∗∗∗*^	.19	.86	7.21^*∗∗∗*^
Regular recovery breaks	.37^*∗∗*^	.12	.78	4.483^*∗∗∗*^
No changes in working hours	.32^*∗∗*^	.11	.76	4.15^*∗∗∗*^
Timely changes to working hours	.28^*∗∗*^	.08	.66	2.96^*∗∗∗*^
Suitable ratio amount versus time	.37^*∗∗*^	.10	.74	3.915^*∗∗∗*^
Time for core tasks	.43^*∗∗∗*^	.08	.70	3.302^*∗∗∗*^
Uniform workload	.29^*∗∗*^	.09	.73	3.74^*∗∗∗*^
No multiple tasks	.32^*∗∗*^	.14	.81	5.19^*∗∗∗*^
No interruptions (from people)	.38^*∗∗∗*^	.12	.78	4.61^*∗∗∗*^
No interruptions (due to ICT)	.35^*∗∗*^	.22	.88	8.66^*∗∗∗*^
Comprehensive information	.54^*∗∗∗*^	.03	.50	1.99^*∗∗*^
Availability of work equipment	.61^*∗∗∗*^	.08	.69	3.242^*∗∗∗*^
*Social relations*				
Respect among colleagues	.65^*∗∗∗*^	.08	.71	3.416^*∗∗∗*^
Support among colleagues	.63^*∗∗∗*^	.10	.75	3.977^*∗∗∗*^
Professional conflict solving	.57^*∗∗∗*^	.09	.71	3.467^*∗∗∗*^
Coordination of joint tasks	.59^*∗∗∗*^	.08	.69	3.244^*∗∗∗*^
Helpful feedback from supervisor	.31^*∗∗*^	.09	.73	3.724^*∗∗∗*^
Acknowledgement from supervisor	.24^*∗*^	.16	.84	6.08^*∗∗∗*^
Respect from supervisor	.49^*∗∗∗*^	.18	.85	6.671^*∗∗∗*^
Support from supervisor as needed	.37^*∗∗*^	.09	.73	3.729^*∗∗∗*^
*Working environment*				
Sufficient Space	.36^*∗∗*^	.14	.81	5.234^*∗∗∗*^
Contact opportunities	.54^*∗∗∗*^	.07	.67	3.032^*∗∗∗*^
Retreat possibilities	.12	.12	.78	4.597^*∗∗∗*^
No unpleasant odours	.46^*∗∗*^	.29	.91	11.73^*∗∗∗*^
Quiet working environment	.27^*∗*^	.25	.90	9.849^*∗∗∗*^
Pleasant climate	.32^*∗∗*^	.28	.91	11.12^*∗∗∗*^
Appropriate lighting	.40^*∗∗∗*^	.20	.87	7.577^*∗∗∗*^
No hazardous/biological agents	.64^*∗∗∗*^	.33	.93	13.92^*∗∗∗*^
No heavy physical loads	.52^*∗∗∗*^	.35	.94	15.54^*∗∗∗*^
Varied postures	.14	.03	.43	1.75^*∗*^

PsyHealth	.42	.15	.77	

*Note*. *N*_activity  groups_ = 22; *N*_mean_ = 27; ^*∗∗∗*^*p* < .001; ^*∗∗*^*p*< .05; ^*∗*^*p*< .01; *ICC*: intraclass correlation; within-group agreement measured at the item level with r_*wg*_.

**Table 2:** Descriptive statistics for the different methods and interrater agreement (IRA).

Work activities companies A to K	Self-ratings			Committee ratings			**IRA**
	*N*	*Mean*	*SD*	*N*	*Mean*	*SD*	***ICC***
*Company A*							
Administrative work A	23	1.99	.56	9 (12)	1.46	.77	**.75**
Childcare	40	2.19	.54	10 (12)	2.48	.92	**.77**
Fire service	27	1.73	.55	7 (12)	1.94	.95	**.72**
Administrative work B	12	2.04	.58	4 (12)	2.47	.83	**.77**
Administrative work C	18	2.41	.52	3(12)	2.52	.81	**.86**
*Company B*							
Pharmaceutical work	54	1.91	.51	2 (3,3)	2.34	.73	**.71**
*Company C*							
Medical-psychological work	10	2.10	.52	4 (3,3,4,6)	1.92	.64	**.77**
Service, kitchen, technology, cleaning work	12	1.98	.52	5 (3,3,3,4,6)	2.20	.60	**.65**
Administrative work	12	2.10	.61	3 (3,4,6)	2.12	.65	**.80**
*Company D*							
Sales work	26	2.12	.65	4 (4,7,7,11)	2.39	.59	**.82**
Production work	19	1.77	.56	5 (4,4,7,7,11)	1.75	.70	**.84**
Laboratory work	12	1.85	.75	4 (4,7,7,11)	2.24	.48	**.67**
Management work	22	2.20	.42	3 (4,7,11)	2.51	.38	**.59**
Administrative work	38	2.25	.47	2 (4,7)	2.05	.62	**.55**
*Company E*							
Law enforcement service	42	1.74	.44	6 (3,5,5,4,6,10)	2.07	.49	**.72**
*Company F*							
Development engineering/customer acquisition	17	2.24	.54	2 (3,11)	2.40	.65	**.78**
Production, service, stock	17	1.92	.55	2 (3,3)	2.58	.52	**.39**
*Company G*							
Childcare	22	2.38	.45	4 (3,3,4,7)	2.25	.73	**.73**
*Company H*							
Security surveillance	97	1.75	.52	6 (1,3,3,4,5,11)	1.59	.54	**.81**
*Company I*							
Physiotherapeutic work	13	2.15	.67	3 (2,4,6)	2.13	.66	**.82**
*Company J*							
Administrative work	20	2.00	.52	3 (4,5,7)	2.49	.57	**.55**
*Company K*							
Administrative work with citizen contact	45	2.07	.58	5 (1,3,4,4,5)	2.26	.62	**.79**

PsyHealth							**.71**

Note. *N*: number of raters; *SD*: Standard Deviation; IRA: interrater agreement; *ICC*: intraclass correlation, *unjust, mean*; 1: management, 2: management representative, 3: supervisor, 4: staff council representative, 5: occupational safety officer, 6: occupational physician, 7: human resource representative, 8: occupational health manager, 9: equal opportunity commissioner, 10: representative of severely handicapped persons, 11: other/not applicable, 12: group assessment, 13: safety representative.

## Data Availability

The statistical data used to support the findings of this study have not been made available due to data protection of the participating companies and their employees. The study center will consider requests for data from researchers who meet the criteria for access to confidential data.
