# Corrigendum to “Comparability of Self-Ratings and Observer Ratings in Occupational Psychosocial Risk Assessments: Is There Agreement?”

**DOI:** 10.1155/2020/6425845

**Published:** 2020-06-12

**Authors:** Isabell Schneider, Martin Mädler, Jessica Lang

**Affiliations:** Teaching and Research Area for Occupational Health Psychology, RWTH Aachen University, Aachen, Germany

In the article titled “Comparability of Self-Ratings and Observer Ratings in Occupational Psychosocial Risk Assessments: Is There Agreement?” [1], References [5, 8] should be removed since they were recommended during the review process and do not contribute essentially to the topic.

The references to be removed are as follows:

[5] N. Mucci, G. Giorgi, M. Roncaioli, J. F. Perez, and G. Arcangeli, “The correlation between stress and economic crisis: a systematic review,” Neuropsychiatric Disease and Treatment, vol. 12, pp. 983–993, 2016.

[8] G. Giorgi, G. Arcangeli, N. Mucci, and V. Cupelli, “Economic stress in the workplace: the impact of fear of the crisis on mental health,” Work, vol. 51, no. 1, pp. 135–142, 2015.

This finding is particularly alarming because employees more frequently report psychosocial risks and strain during times of economic recession [6]. For instance, insomnia ratings were greater among nurses who experienced a pay cut than among nurses whose payment conditions had not changed [7]. If supervisors were trained in interactional justice (i.e., an intervention aimed at improving psychosocial working conditions), the degree of insomnia and thus the individual strain response decreased faster than those for nurses whose supervisors did not receive a training. Thus, the assessment of psychosocial risks during crisis time appears to be a strategic topic [6].
